# Supplementary material for: Design and Optimization of A Magneto-Plasmonic Sandwich Biosensor for Integration within Microfluidic Devices
Source: Biosensors (Basel). 2022 Sep 27;12(10):799. doi: 10.3390/bios12100799 (PMC9599771; doi:10.3390/bios12100799)
Supplement: Supplementary file 1 [file biosensors-12-00799-s001.zip › biosensors-1922020-supplementary.pdf]

biosensors

MDPI

Supplementary Material

Design and Optimization of A Magneto-Plasmonic Sandwich Biosensor for Integration within Microfluidic Devices

Mona Soroush [1,2], Walid Ait Mammar [1], Axel Wilson [1], Hedayatollah Ghourchian [2], Michèle Salmain [3,*] and Souhir Boujday [1,*]

[1] Sorbonne Université, CNRS, Laboratoire de Réactivité de Surface (LRS), F-75005 Paris, France
[2] Laboratory of Bioanalysis, Institute of Biochemistry & Biophysics, P.O. Box: 13145-1365, University of Tehran, 1417614335 Tehran, Iran
[3] Sorbonne Université, CNRS, Institut Parisien de Chimie Moléculaire (IPCM), F-75005 Paris, France
* Correspondence: michele.salmain@sorbonne-universite.fr (M.S.); souhir.boujday@sorbonne-universite.fr (S.B.)

Quantification of Antibody-to-AuNP Ratio

FITC-labeled goat anti-rabbit IgG (12 μg/mL; 5 mL) was thiolated with Traut's reagent according to 2.7. After the blocking step with BSA, AuNP bioconjugate was washed four times with 5 mL 10 mM phosphate buffer pH 7.4 (13,000 rpm, 30 min). The four supernatants named supernatants 1 to 4 were collected and their fluorescence spectra recorded (Figure S1b). The concentration of unbound FITC-labeled Ab was calculated from the calibration plot established from FITC-labeled Ab standard solutions (Figure S1a).

The concentration of FITC-labeled Ab in supernatants 1–4 is 5.33, 0.36, 0.24 and 0.07 μg/mL, respectively. By subtraction, the concentration of FITC-Ab bound to AuNP is 12 – (5.33 + 0.36 + 0.24 + 0.07) = 6 μg/mL.

The number of antibody molecules per AuNP equals the concentration of bound Ab (in molar unit) divided by the concentration of AuNP (in molar unit) = 39.4 nM/2.8nM = 14

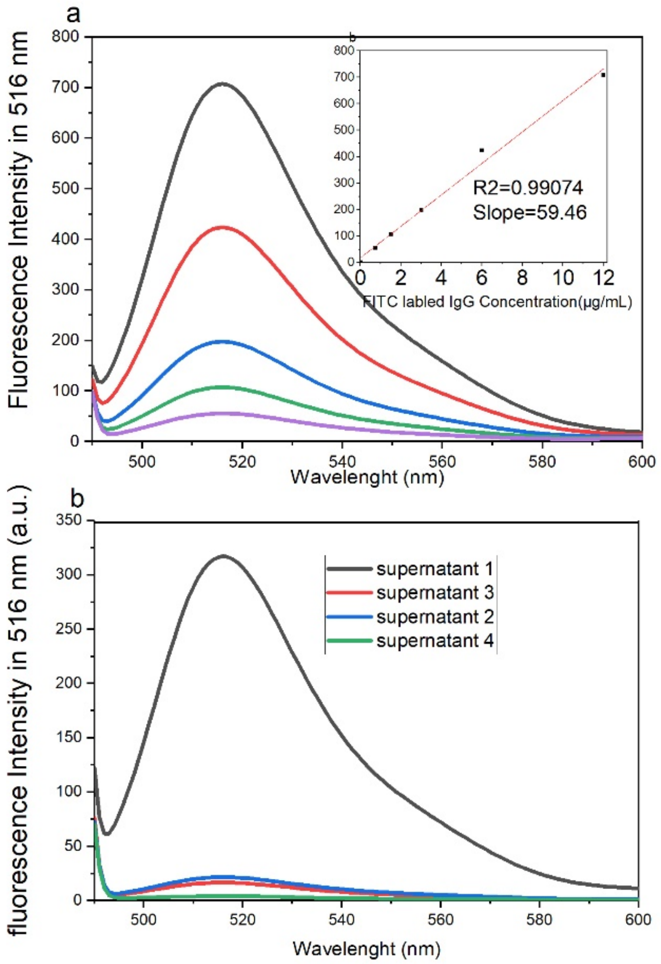

Figure S1. (**a**) Fluorescence spectra of standard solutions of FITC-labeled goat anti-rabbit IgG (Ab) in PB; inset: calibration plot established from the emission intensity at 516 nm (λ_{ex} = 485 nm). (**b**) Fluorescence spectra of supernatants 1–4.

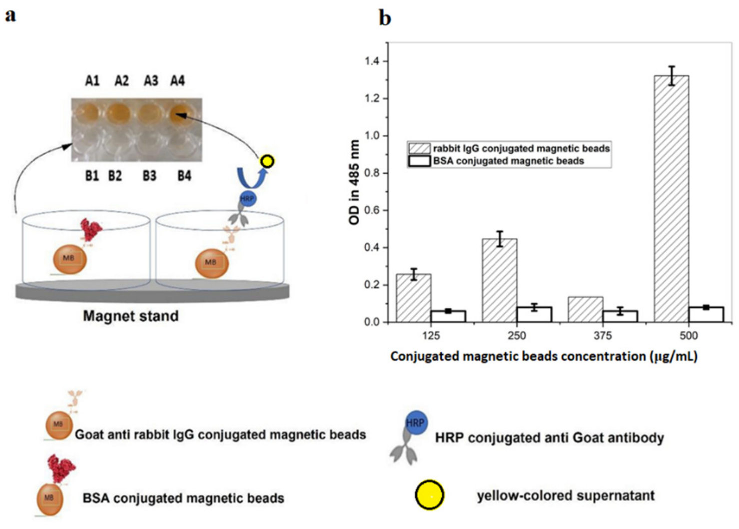

Figure S2. ELISA of MB-Ab. (**a**) MB-Ab or MB-BSA (negative control) are immobilized by a magnet stand at the bottom of wells of a microplate; HRP-labeled anti-goat IgG is added to the wells followed by addition of a colorless substrate (OPD + H_2O_2) that is converted to a yellow product; supernatants are transferred to wells A1–A4 (MB-Ab) or B1–B4 (MB-BSA) for OD_{485nm} measurement. (**b**) Optical density (OD_{485nm}) of wells A1–A4 and B1–B4.

Data processing for Direct Detection with the Standard Cell

Since only magnetic beads absorb at 450 nm and the same concentration of the magnetic beads was utilized for all samples, all the curves were shifted to match 1 a.u. at 450 nm in Figure 6a. The first derivatives shown in inset 6a were calculated with a Savitzky–Golay filter using a 5th order polynomial and a 200 points window for a total of 1905 points per spectrum.

The 475–585 nm spectral range was chosen to integrate the area comprised between a strait, tangent line and the signal as shown in Figure S3 below. The calculated area was directly proportional to the concentration of the target and the dose–response curve displayed in Figure 6b was established.

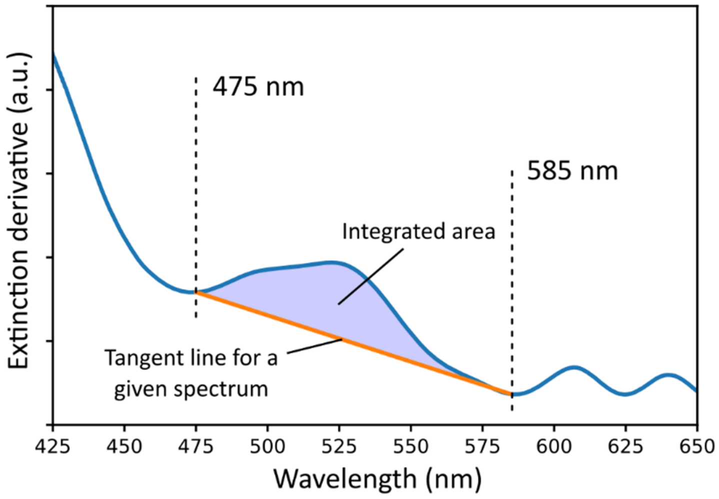

Figure S3. Data processing for the direct detection with the standard cell.

Data processing for the indirect detection (standard cell; Figure 7 and μ-fluidic cell; Figure 9)

To account for a shift observed using the Insplorion spectrometer, all the spectra were shifted to 0 at 750 nm where no species absorbs. Using this method, the maximum extinction at 530 nm was indirectly proportional to the concentration of the target and the dose–response curves depicted in Figure 7b and Figure 9b were established.
